# COVID-19 Outbreak in a Large Penitentiary Complex, April–June 2020, Brazil

**DOI:** 10.3201/eid2703.204079

**Published:** 2021-03

**Authors:** Fernando A. Gouvea-Reis, Patrícia D. Oliveira, Danniely C.S. Silva, Lairton S. Borja, Jadher Percio, Fábio S. Souza, Cássio Peterka, Claudia Feres, Janaína de Oliveira, Giselle Sodré, Wallace dos Santos, Camile de Moraes

**Affiliations:** Ministério da Saúde, Brasília, Brazil (F.A. Gouvea-Reis, P.D. Oliveira, D.C.S. Silva, L.S. Borja, J. Percio, C. de Moraes);; Secretaria de Estado de Administração Penitenciária, Brasília (F.S. Souza);; Secretaria de Estado da Saúde do Distrito Federal, Brasília (C. Peterka, C. Feres, J. de Oliveira, G. Sodré, W. dos Santos)

**Keywords:** Brazil, coronavirus disease, COVID-19, infectious diseases, outbreaks, overcrowding, prisons, public health, SARS-CoV-2, severe acute respiratory syndrome coronavirus 2, underlying medical conditions, viruses, zoonoses

## Abstract

An outbreak of coronavirus disease began in a large penitentiary complex in Brazil on April 1, 2020. By June 12, there were 1,057 confirmed cases among inmates and staff. Nine patients were hospitalized, and 3 died. Mean serial interval was ≈2.5 days; reproduction number range was 1.0–2.3.

Detention facilities constitute an environment optimal for the introduction and spread of respiratory infectious diseases. Living conditions of inmates are frequently overcrowded and poorly ventilated, might provide limited access to running water, and lack adequate sanitary facilities. These conditions increase risk factors for and background prevalence of infection (*1*,*2*). Furthermore, prisons commonly must deal with understaffing and lack of resources, presenting additional challenges to triaging and treating higher-risk patients in a timely manner (*3*). 

The first case of coronavirus disease (COVID-19) in Brazil was reported on February 26, 2020. The introduction of severe acute respiratory syndrome coronavirus 2 (SARS-CoV-2), the causative agent of COVID-19, into prisons adds an extra burden into an already overwhelmed environment. COVID-19 could be introduced through staff, visitors, or inmates under a semi-open regime, in which they spend the day outside the penitentiary complex and return in the evening. In addition, a new disease for which there is no existing immunity increases the health risk. Therefore, control of COVID-19 in prisons must be considered an essential part of a public health response (*4*). 

In Brazil, ≈750,000 people are imprisoned in a system built to hold just over 442,000 (*5*). By June 12, Brazil had reported 828,810 COVID-19 cases nationwide, with >2,200 of those within prison settings (*6*,*7*). Testing in Brasília, located in Brazil’s Federal District, accounted for 65.5% of all COVID-19 tests performed among imprisoned persons; ≈48% of the total national cases in prisons were reported in a maximum-security penitentiary complex in Brasília. The complex includes 4 prison units: Unit I houses persons under pretrial detention, Unit II houses inmates under a semi-open regime, and Units III and IV house convicted inmates. Overall, the prison is one of the largest penitentiary complexes in Brazil, housing >13,000 male inmates, as of June 2020. 

In this report, we provide a descriptive analysis of the outbreak and estimate the disease transmissibility in its early stages. Data were collected from secondary sources, including the penitentiary monitoring dataset for COVID-19 notifications, the penitentiary administration system, and the monitoring resources of the healthcare system. The public health response was a joint effort from the state health and security departments, local health and security teams, and the Brazil Ministry of Health’s Brazilian Field Epidemiology Training Program. Ethics approval was obtained under CONEP (Comissão Nacional de Ética em Pesquisa [National Research Ethics Commission], protocol number 37007220.1.0000.0008). 

## The Study

The first COVID-19 case in Brasília was reported on March 5; on April 1, the first case in the penitentiary complex, in a prison guard, was confirmed. The earliest infections occurred among security officers; the first inmate with COVID-19 was reported on April 7 in Unit I, the pretrial detention area. Six cases were reported on April 10 in 2 different wings from the same block in that unit. The location of the first cases in the other prison units indicated that COVID-19 had dispersed across the penitentiary complex. In Unit II, 13 cases were reported on April 9–10 in 3 different blocks. In Unit III, 5 cases were confirmed on April 17 in 2 different blocks. In Unit IV, 6 cases were confirmed on April 15–17 in 3 different blocks. Although the virus was not introduced inside the penitentiary complex until ≈1 month after the first reported case in Brasilia, the complex rapidly attained the highest incidence in the region. By May 1, whereas the incidence rate was 47 cases/100,000 persons in the city, it was 1,832 cases/100,000 persons among inmates (*8*). 

**Figure 1:**
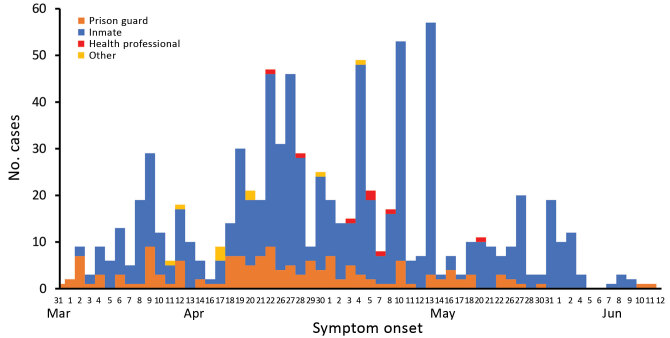
Distribution of symptomatic coronavirus disease cases over time by symptom onset date in a penitentiary complex, Brasília, Brazil, March–June 2020.

During April 1–June 12, there were 1,057 reported cases at the prison: 859 (81.3%) in inmates, 180 (17.1%) in prison guards, 9 (0.8%) in contracted staff, and 9 (0.8%) in health professionals. Distribution of the symptomatic cases over time is shown in Figure 1. Nine patients were hospitalized, and 3 deaths were reported: 1 prison guard and 2 inmates. 

Among infected inmates, mean age was 38 years (SD 14.1 years); 296 (34.5%) were 18–29 years of age, 245 (28.5%) 30–39 years, 124 (14.4%) 40–49 years, 61 (7.1%) 50–59 years, and 133 (15.5%) >60 years. Information about ethnicity was available for 783 patients; 407 (52.0%) were mixed race, 214 (27.3%) White, 93 (11.9%) Asian, 67 (8.5%) Black, and 2 (0.3%) Indigenous. Underlying medical conditions were reported in 160 (18.6%) patients; the most prevalent were cardiovascular diseases (11.8%), diabetes (5.1%), and pneumopathies (2.8%). We were able to evaluate the presence of symptoms on medical records from 401 patients with confirmed COVID-19 cases. The most prevalent symptoms were headache (34.9%), cough (30.2%), and fever (28.9%) (Table). 

**Table:** Characteristics of coronavirus disease cases within the penitentiary complex, Brasília, Brazil, April–June 2020

Characteristic	No.	% Total
Age, y		
18–29	296	34.5
30–39	245	28.5
40–49	124	14.4
50–59	61	7.1
>60	133	15.5
Ethnicity*		
Asian	93	11.9
White	214	27.3
Indigenous	2	0.3
Mixed race	407	52.0
Black	67	8.5
Symptoms†		
Headache	140	34.9
Cough	121	30.2
Fever	116	28.9
Ageusia/anosmia	79	19.7
Dyspnea	67	16.7
Myalgia	42	10.5
Sore throat	32	8.0
Nasal congestion	23	5.7
Diarrhea	16	4.0
Underlying health conditions		
Cardiovascular disease	101	11.8
Diabetes	44	5.1
Pneumopathies	24	2.8
Others‡	8	0.9

*Data available for 783 cases.  †Data available for 401 cases.  ‡Includes metabolic disorders, immunosuppression, psychiatric disease, and chronic hepatitis.

We applied the EpiEstim R package (https://cran.r-project.org) to estimate the reproduction number over time (R_t_), the number of secondary PCR-confirmed cases resulting from a single initial case, for the penitentiary complex. We estimated the serial interval by computing the difference between the dates of symptom onset for pairs of primary and secondary infected inmates in 144 cases confirmed by reverse transcription PCR (RT-PCR) recorded in April, during the early stages of the outbreak, within all 4 prison units. We identified the primary infected inmate as the person in a cell having the first RT-PCR–confirmed case; we identified secondary infected inmates as anyone sharing a cell with a primary case patient who tested positive for COVID-19 by RT-PCR <14 days after symptom onset in the cellmate. Local health teams identified suspected cases through daily active case finding; patients with confirmed cases were isolated as separate cohorts. 

**Figure 2:**
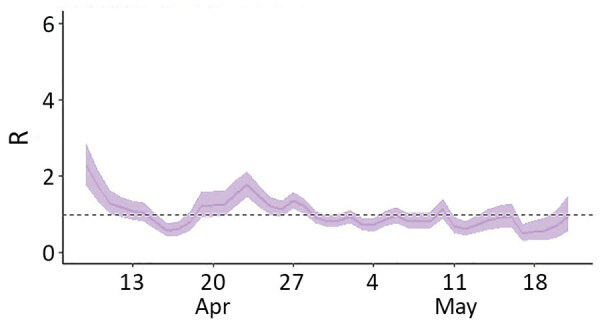
R_t_ for severe acute respiratory syndrome coronavirus 2 transmission in a penitentiary complex, Brasília, Brazil, April–May 2020. Blue line indicates median R_t_; blue shading indicates 95% CI. Dashed line indicates R_t_ = 1.

We estimated the mean serial interval at 2.51 days (SD 1.21). We found high transmissibility at the start of the outbreak, when the overall R_t_ was 2.28 in the prison complex (Figure 2). April was the month with the most intense transmission; Units III and IV reported 149 cases during April 20–30, which is reflected in the R_t_ peaks in those units (Appendix Figure). R_t_ decreased over time, to ≈1.0 during most of May. 

## Conclusions

We found a shorter serial interval for COVID-19 in this prison than that estimated for Brazil overall (*9*). This finding supports the idea of a faster viral spread in overcrowded settings, and considering that the estimated serial interval was lower than the mean incubation period, the likely transmission of presymptomatic cases might have played an important role in viral spread inside the prison complex (*10*,*11*). Asymptomatic or presymptomatic cases, other sources of infection, or inmates failing to report symptoms might have affected accurately identifying primary infected inmates, resulting in our possibly underestimating the serial interval, R_t_, or both. Considering the overcrowded conditions in the penitentiary complex and the impossibility of mandating effective social distancing, implementing broad testing strategies is fundamental for accurately measuring viral spread and planning better interventions. 

Appendix COVID-19 outbreak in a large penitentiary complex, April–June 2020, Brazil.
