# Supplementary material for: COVID-19 Outbreak in a Large Penitentiary Complex, April–June 2020, Brazil
Source: Emerg Infect Dis. 2021 Mar;27(3):924–7. doi: 10.3201/eid2703.204079 (PMC7920649; doi:10.3201/eid2703.204079)
Supplement: Appendix — COVID-19 outbreak in a large penitentiary complex, April–June 2020, Brazil. [file 20-4079-Techapp-s1.pdf]

Article DOI: https://doi.org/10.3201/eid2703.204079

COVID-19 Outbreak in a Large Penitentiary Complex, April–June 2020, Brazil

Appendix

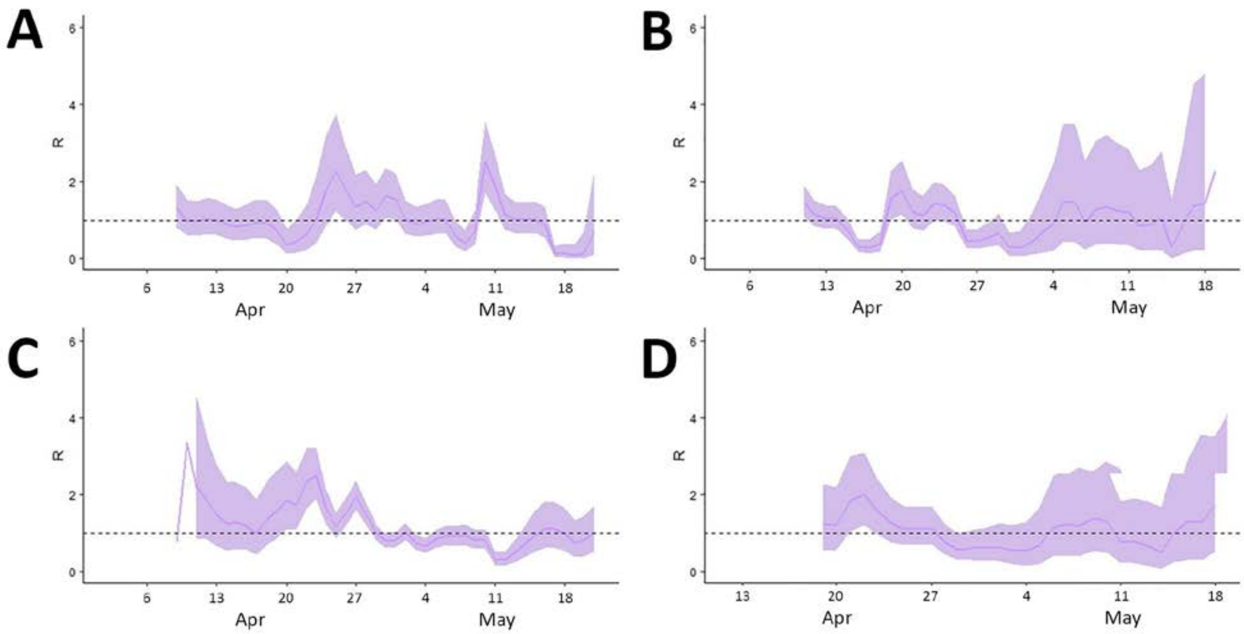

Appendix Figure. SARS-CoV-2 reproduction number over time by prison unit. Penitentiary complex, Brasília, DF, April–May 2020. A) Unit I; B) Unit II; C) Unit III; D) Unit IV. Blue line is median Rt; blue shadow is 95% CI.
